# Native amphibian toxin reduces invasive crayfish feeding with potential benefits to stream biodiversity

**DOI:** 10.1186/s12862-023-02162-6

**Published:** 2023-09-13

**Authors:** Gary M. Bucciarelli, Sierra J. Smith, Justin J. Choe, Phoebe D. Shin, Robert N. Fisher, Lee B. Kats

**Affiliations:** 1 Department of Ecology and Evolutionary Biology, University of California, Los Angeles, Los Angeles, CA 90095 USA; 2 Department of Wildlife, Fish, and Conservation Science, University of California, Davis, Davis, CA 95616 USA; 3 Natural Science Division, Pepperdine University, Malibu, CA 90263 USA; 4 Western Ecological Research Center, U.S. Geological Survey, San Diego, CA 92101 USA

**Keywords:** Santa Monica Mountains, *Taricha torosa*, Crayfish, *Procambarus clarkii*, Tetrodotoxin

## Abstract

**Background:**

Biodiversity is generally reduced when non-native species invade an ecosystem. Invasive crayfish, *Procambarus clarkii*, populate California freshwater streams, and in the Santa Monica Mountains (Los Angeles, USA), their introduction has led to trophic cascades due to omnivorous feeding behavior and a rapid rate of population growth. The native California newt, *Taricha torosa*, possesses a neurotoxin, tetrodotoxin (TTX), that affects freshwater animal behavior. Given *P. clarkii* has a limited evolutionary history with TTX, we hypothesized that TTX may affect crayfish feeding behaviors. To determine if TTX affects *P. clarkii* behavior, we measured cumulative movement and various feeding behaviors of *P. clarkii* exposed to (i) waterborne, ecologically realistic concentrations of TTX (~ 3.0 × 10^− 8^ moles/L), (ii) an anuran chemical cue to account for intraguild cues, or (iii) a *T. torosa* chemical cue with quantitated TTX in it (~ 6.2 × 10^− 8^ moles/L).

**Results:**

We found that the presence of TTX in any form significantly reduced crayfish movement and decreased the amount of food consumed over time. Crayfish responses to the anuran treatment did not significantly differ from controls.

**Conclusion:**

Our laboratory results show that naturally occurring neurotoxin from native California newts limits invasive crayfish foraging and feeding rates, which may play a role in preserving local stream ecosystems by limiting invasive crayfish behaviors that are detrimental to biodiversity.

**Supplementary Information:**

The online version contains supplementary material available at 10.1186/s12862-023-02162-6.

## Introduction

The importance of biodiversity is increasingly apparent as studies continue to indicate that the quality and quantity of species diversity within a community are essential to ecosystem functions such as the regulation of atmospheric gaseous composition, climate, waste assimilation, and pollution control [1]. Biodiversity loss reduces ecosystem productivity and stability, dampening the functioning of ecosystems and organisms [2, 3]. Previous experiments have shown that more diverse communities provide higher ecosystem functioning overall compared to less diverse communities, and recent field experiments have corroborated those findings by showing biodiversity loss in natural ecosystems has a strong effect on functioning [4]. One of the leading factors of biodiversity loss is the spread of invasive species [5], which has increased in recent decades due to inadvertent dispersal by humans and their expansion driven by climate change [5, 6]. The proliferation of invasive species has raised sustainability and biodiversity concerns due to their net negative ecological, biological, and environmental effects [7, 8]. Invasive species are known to threaten biodiversity through predation, competition, displacement, and hybridization among other influences [9] because they tend to rapidly populate an ecosystem [10] with broad ecological consequences, such as the disruption of trophic relationships that naturally regulate human disease vectors [11].

The vulnerability of an ecosystem to an invasion, and therefore biodiversity loss, is dependent on a multitude of biotic and abiotic factors [12]. Disturbance is an influential abiotic factor that can affect whether an introduced species becomes established in a community, and disruption of normal ecosystem functioning is a catalyst for non-native invasion with a strong correlation between invasive species success and disturbances [13]. Freshwater ecosystems are particularly vulnerable communities because of the sensitivity of freshwater organisms such as amphibians and macroinvertebrates (taxa in the orders Ephemeroptera, Plecoptera, and Trichoptera taxa, EPT) that are often utilized as bioindicators of ecosystem integrity [14].

Bioindicators such as benthic macroinvertebrates (BMI) often serve as indicators of stream health due to their sensitivity towards environmental disturbances, narrow and known environmental tolerances, and ease in identification, which allows for their relative abundance and richness to serve as a reflection of the environment [15, 16]. Amphibians are another indicator of stream health and are known to be sensitive to disturbance and environmental change [17], likely because of their biphasic life strategy, but also their increased vulnerability to contaminants. For example, frogs (*Rana esculenta*) absorb atrazine at a rate 300 times greater than mammals [18] .

**Fig. 1:**
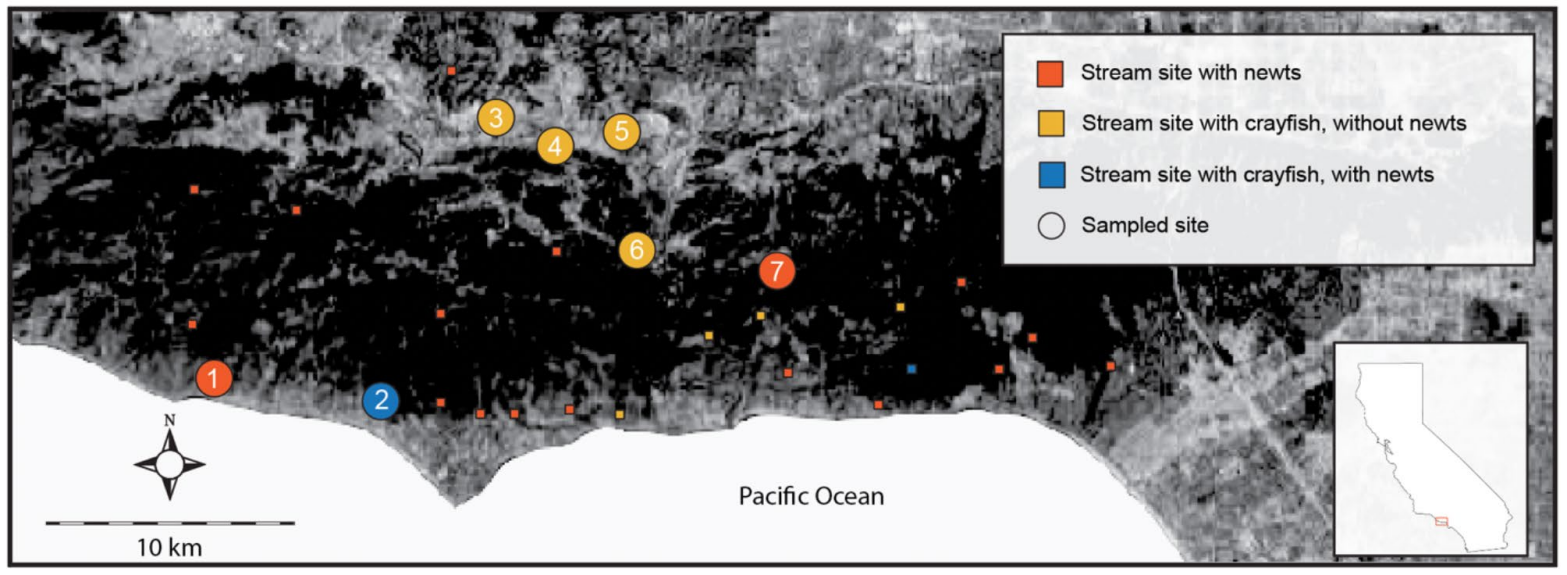
Stream study sites throughout the Santa Monica Mountains. Stream sites sampled as a part of the study are shown with circles; smaller squares represent other stream sites to illustrate the current distribution patterns of newts and crayfish. Sites are: (1) Arroyo; (2) Trancas; (3) Upper Lindero; (4) Lower Lindero; (5) Medea; (6) MLV; (7) Cold Creek. Note, there are only two sites where newts and crayfish presently coexist

In the Santa Monica Mountains of Southern California (Los Angeles, USA, Fig. 1), invasive species are found in a subset of local streams, thereby affecting a variety of native aquatic species [19]. Amphibian population declines are well documented across Southern California, and studies suggest that invasive species heavily contributed to these declines [20, 21]. First documented in Southern California in 1924, the omnivorous red swamp crayfish, *Procambarus clarkii*, has proliferated in numerous Southern California watersheds and invaded streams within the Santa Monica Mountains [22, 23]. *Procambarus clarkii* is a freshwater species native to the southeastern parts of the United States that now inhabits all continents except Antarctica and Australia. The widespread use of *P. clarkii* as fishing bait, its behavioral plasticity, omnivorous feeding behavior, and high reproductive rate have made it one of the most widely distributed freshwater crayfish in the world. In addition to predation on local species, *P. clarkii* is also recognized as an invader capable of reshaping ecosystems [24].

The presence of invasive *P. clarkii* directly relates to the reduced abundance or extirpation of native amphibians [23] that has led to food web impairment and is correlated with local population declines of native tree frogs (*Pseudacris cadaverina, P. regilla*) and California newts (*Taricha torosa*) [7]. The California newt has historically inhabited numerous freshwater streams in the Santa Monica Mountains, but following the introduction of *P. clarkii*, breeding amphibian populations were severely reduced and some extirpated [23, 25]. California newts are currently recognized as a Species of Special Concern in Southern California (Monterey County and south [26], a state-recognized designation that denotes a species, subspecies, or distinct population warrants conservation action(s). Newts (Salamandridae), including the genus *Taricha*, possess a powerful neurotoxin, tetrodotoxin (TTX). During all life stages *T. torosa* have TTX, from egg to adult, and the toxin has also been detected in egg mass material. Breeding adults appear to give off TTX in their aquatic habitat [27] and it is detectable at 1–9 cm in the water around adults at nanomolar concentrations (i.e. 1–10 × 10^− 9^ moles/L; D. Schar, unpublished data). Presumably TTX is also introduced into aquatic breeding habitat from egg masses, as embryos emerge, and while larvae develop. In general, TTX is thought to serve as a chemical defense against predators [28]. However, the neurotoxin also acts as a chemical cue that elicits behavioral responses in vertebrates and invertebrates with consequence to trophic interactions. For example, conspecific interactions between cannibalistic adults and larvae are mediated by TTX [29, 30] and native predators of *T. torosa* larvae, such as dragonfly nymphs (genus *Aeshna*) exhibit impaired predatory feeding behavior when exposed to waterborne TTX [27]. In other systems, TTX serves as a sexual attractant for pufferfish [31, 32] and stimulates snail feeding behavior [33]. Remarkably, despite the toxicity of TTX, California newt adults, egg masses, and larvae are still attacked and consumed by invasive *P. clarkii*, which appears to be due to resistance that arose prior to the major diversification of the currently recognized crayfish super families [34].

Water-borne chemical cues relay crucial information to aquatic metazoans that induce reactions such as predator-avoidance and alarm behaviors [30]. Olfaction guides various aspects of crayfish life strategy, such as predator avoidance and agonistic interactions. As such, chemical cues are a critical source of information for crayfish [35]. Crayfish naturally encounter turbid waters and low light that would make them dependent on non-visual information including chemical cues and tactile sensory information from antennae [36]. Studies show exposure to water from other male conspecifics induces agonistic behavior [37] along with reception and integration of chemical cues from other crayfish [38]. For example, crayfish (*P. clarkii*) that were subjected to several stressors, including social interactions and different environmental variables exhibited anxiety-like behavior, similar to that observed with vertebrates [39]. However, it remains unclear whether TTX from newts in streams might affect crayfish behavior as a waterborne chemical cue.

In the Santa Monica Mountains, crayfish and California newts do coexist in two streams, largely due to geology and flooding dynamics of these sites that promote flooding during heavy rainstorms, which ultimately reduces the number of crayfish [21]. However, TTX that effuses from newts, larvae, or eggs into streams may act as a chemical cue that alters crayfish behavior, or it may have sublethal physiological impacts that modify behaviors, and these effects may reduce foraging behavior. Other local studies have demonstrated that waterborne TTX from California newts limits the dispersal of an invasive snail, the New Zealand mud snail (*Potamopyrgus antipodarum*), reduces the movement and strike velocities of native invertebrate predators (Anisoptera, *Aeshna spp*), and alters foraging patterns of the stream benthic invertebrate community in habitat where there are adult newts [27, 40]. In this study, we use laboratory experiments to test whether waterborne TTX and chemical cue solutions from newts or sympatric frogs affect the movement and feeding behavior of *P. clarkii*. We then assess the relevance of these laboratory results in the field by qualitatively evaluating biodiversity patterns in crayfish-invaded streams where newts are present and absent. Collectively, our results show that TTX has strong ecological impacts on crayfish behavior.

## Results

### Movement bioassays

**Fig. 2:**
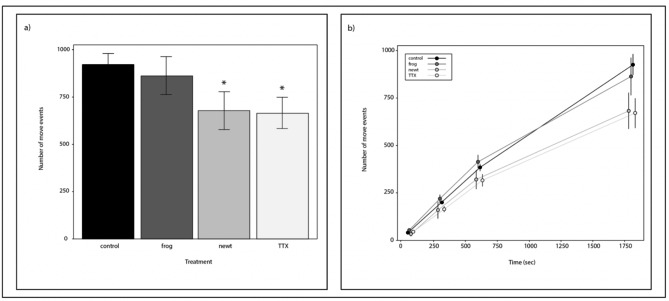
The effect of amphibian chemical cues and waterborne TTX on crayfish movement. (**a**) The number of instances a crayfish moved was significantly reduced in the presence of newt chemical cues (GLMM: p < 0.01) and TTX (GLMM: p < 0.01) treatments relative to the control. The same response was not observed when crayfish were exposed to tree frog chemical cues (p = 0.95). Asterisks show statistically significant treatments relative to the control. (**b**) Average movement of crayfish during 30-minute trials at four discrete time points shows crayfish continued to move throughout the experiment, but overall movement patterns differ as early as three minutes into the experiment. By the end of the experiment the cumulative number of moves a crayfish made differed between the newt (p < 0.01) and TTX (p < 0.01) treatments relative to the control, but no other pairwise comparisons were significant. Error bars represent the s.e.m

Laboratory bioassays were performed to test whether crayfish foraging behavior was affected by a solution of waterborne TTX at ecologically realistic concentrations (3.0 × 10^− 8^ mol/L), a chemical cue solution from *T. torosa* that contained TTX (6.2 × 10^− 8^ mol/L), and a chemical cue from a sympatric tree frog (*P. regilla*). We found that crayfish movement significantly differed in treatments with the newt chemical cue and TTX solutions (TTX: 𝛽 = -0.31, t = -2.81, p < 0.01; newt: 𝛽 = -0.34, t = -3.05, p < 0.01) relative to the control, but there was no significant difference between the control and the frog chemical cue treatment (frog: 𝛽 = -0.01, t = -0.05, p = 0.95). When we recoded the model to make comparisons among all treatments, only the TTX (p = 0.01) and newt (p = 0.01) treatments differed from the control (Fig. 2a). Over the duration of the experiment, crayfish moved less on average in the TTX ($\overline{x }$= 668, s.e.m. = ± 80.4, 95% CI = 379 (1046 − 290)) and newt chemical cue treatments ($\overline{x }$= 681, s.e.m. = ± 97.2, 95% CI = 597 (1278–84)) compared to the frog chemical cue ($\overline{x }$= 863, s.e.m. = ± 100.3, 95% CI = 691 (1555 − 172)) and control ($\overline{x }$= 924, s.e.m. = ± 56.8, 95% CI = 522 (276–494)) (Fig. 2b).

### Feeding bioassays

**Fig. 3:**
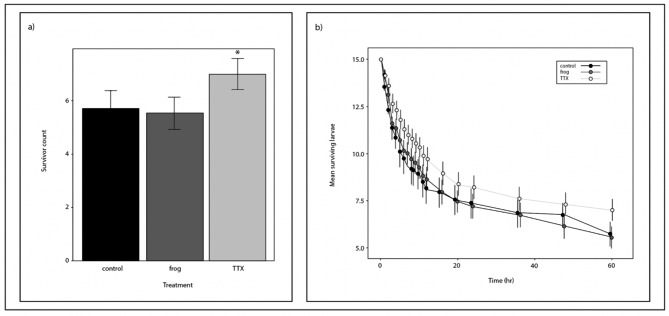
Mosquito larval survivorship is greatest in the presence of crayfish predators when TTX is present. (**a**) The number of surviving larvae was affected by the presence of TTX (GLMM, p < 0.001) and relative to the control, crayfish feeding was reduced only in the TTX treatment. Asterisks show statistically significant treatments relative to the control. (**b**) Initial counts of larvae (15 per replicate) steadily declined from 0 to 60 h, but survivorship was significantly greater with waterborne TTX. Regular intervals of data collection are shown as points with s.e.m. bars, comprising 20 trials per treatment. Connecting lines illustrate the decline overtime

We performed laboratory feeding experiments to test if TTX affects crayfish feeding. The bioassays included a TTX solution (3.0 × 10 ^−8^ mol/L) and a sympatric tree frog chemical cue solution. Relative to the control, the number of surviving mosquito larvae significantly differed in the TTX treatment (𝛽 = 0.12, t = 5.2, p < 0.01), but not the frog cue treatment (𝛽 = 0.02, t = 1.2, p = 0.23). Statistical comparisons of the number of surviving larvae by treatment detected significant differences between the TTX treatment and the control (p < 0.01) and between the TTX and frog chemical cue treatments (p < 0.01), but not between the frog chemical cue treatment and the control (p = 0.62) (Fig. 3a). Over time, fewer numbers of larvae survived in the frog chemical cue treatment and control (frog: $\overline{x}$ = 5.5, s.e.m. = ± 0.59, 95% CI = 2.4 (3.1–7.9); control: $\overline{x}$ = 5.7, s.e.m. = ± 0.67, 95% CI = 2.4 (3.2–8.2)) compared to the TTX treatment ($\overline{x}$ = 7.0, s.e.m. = ± 0.59, 95% CI = 3.06 (3.9–10.0)) (Fig. 3b).

### Field assessments

To better understand the potential ecological realism of the laboratory experiments, we quantified stream benthic macroinvertebrate (BMI) biodiversity in crayfish invaded streams where newts co-occur, crayfish invaded streams without newts, and streams with newts, where crayfish have never been introduced. The analyses of community diversity and derived metrics indicated that sites with newts and no crayfish (Arroyo and Cold Creek) had greater biodiversity than streams with crayfish and no newts (Upper and Lower Lindero, Medea, and MLV) (Table 1). However, the stream where newts and crayfish coexist (Trancas) had biodiversity metrics all greater than crayfish only sites and relatively similar or higher scoring metrics compared to sites with newts only. At the coexistence site, counts of invertebrates, taxonomic richness, evenness, sensitive taxa counts (EPT), and biological integrity metrics were greater relative to crayfish only sites, and some metrics were even higher than sites with newts only (metrics defined in [15]). We found that the Southern California Index of Biological Integrity (SC-IBI) was roughly 3-fold greater at the coexistence site than sites with crayfish only, and although it is roughly half relative to one of our non-crayfish sites (Cold Creek), the score at Trancas exceeded that of the other non-crayfish stream (Arroyo). Similarly, scores of the California Stream Condition Index (CSCI), which factors abiotic landscape features into assessments of BMI to derive scores based on observed versus expected biodiversity, were approximately 25 − 50% lower in the crayfish only streams relative to the coexistence site, comparable between Arroyo and Trancas, and greatest at the other site without crayfish.

**Table 1:** Assessment of benthic macroinvertebrate communities in streams with crayfish (upper and lower Lindero, Medea, MLV), with newts (Arroyo, Cold Creek), or where there is coexistence (Trancas). Counts of species assigned to a functional group are also provided and include species counts for taxa that classify as intolerant, predator, scraper, shredder, burrower, climber, clinger, or swimmer (see [15, 41] for group definitions)

	Upper Lindero	Lower Lindero	Medea	MLV	Trancas	Arroyo	Cold Creek
Count	139	283	396	168	515	157	503
Taxonomic richness	10	11	9	6	17	16	30
Intolerant	0	0	0	0	1	3	5
Predator	2	3	1	1	6	4	12
Scraper	2	2	2	1	2	0	4
Shredder	0	0	0	0	0	1	3
Burrower	1	1	2	1	2	2	6
Climber	1	1	0	1	1	2	3
Clinger	1	1	0	0	3	4	7
Swimmer	1	2	1	2	4	1	5
EPT Taxa	1	1	1	2	4	5	11
Ephemeroptera	1	1	1	2	2	2	4
Plecoptera	0	0	0	0	1	2	3
Trichoptera	0	0	0	0	1	1	4
Shannon-Weaver	1.27	1.66	0.84	1.51	1.76	2.1	2.7
SC-IBI	14.9	14.9	12.1	17.8	43.5	32.1	76.4
CSCI	0.54	0.57	0.39	0.42	0.71	0.7	0.93

## Discussion

The disruption of aquatic biodiversity by invasive species is well documented but few studies have attempted to document whether the presence or absence of a native species can modify or alter the impacts invading species have to biodiversity. We found that waterborne chemical cues from a native amphibian reduce the foraging behaviors of an invasive predator. Our results show that TTX at ecologically relevant concentrations, ranging from 3.0 to 6.2 × 10^− 8^ mol/L, significantly influences crayfish (*P. clarkii*) feeding and movement behavior. The results of the movement bioassays indicate that crayfish respond to TTX and TTX-laden newt chemical cues (Fig. 2) and that the toxin reduces their feeding behavior at these concentrations (Fig. 3). The model results from the feeding analyses showed that the effect size was 6-fold greater in the newt cue treatment (𝛽 = 0.12) compared to the frog treatment (𝛽 = 0.02) highlighting a strong negative effect that TTX can have on crayfish predation. In general, we observed that crayfish feeding behavior decreased over time and trends show that crayfish ate fewer prey in the newt cue treatment through the duration of the trials compared to all other treatments (P < 0.01). The similar behavioral results between the TTX and newt chemical cue treatments imply that crayfish respond to newt chemical cues because of TTX, a potent neurotoxin known to elicit behavioral change in vertebrates and invertebrates. The significant differences in crayfish responses between the frog chemical cue and the TTX and TTX-laden newt chemical cue treatments provide evidence that behavior is specifically affected by TTX from newts, not amphibian-related cues individually or possibly residual citrate from the TTX solution. Other studies have documented that crayfish respond to amphibian cues, but these studies only include amphibian predators such as hellbenders (*Cryptobranchus alleganiensis*), which are known to prey on crayfish [42]. Because TTX and newt cue treatments were the only statistically significant groups in the experiments, our results generally suggest that TTX from newts could alter the behavior of invasive crayfish in pools throughout breeding sites.

In stream ecosystems, chemical cues critically impact the dynamics of freshwater communities [43] and interspecific chemical cues can play a crucial role in community assembly and interspecific ecological processes [44]. For example, seaweed (*Dictyota bartayresii*) produces pachydictyol-A, a type of diterpene alcohol that deters fish feeding [45] and red alga, *Delisea pulchra*, produces halogenated furanones that affect the colonization of bacteria on its surface by interfering with bacteria motility [46]. Similar studies have also documented ways that chemical cues may disrupt freshwater trophic interactions by modifying foraging behavior. For example, freshwater snails (*Physella gyrina*) will modulate their use of cover based on predator or conspecific alarm cues [43]. In our focal system, we have documented behavioral responses of native and invasive stream community members elicited by waterborne TTX and newt chemical cues bearing TTX [27, 40]. Specific to invasive crayfish, chemical cues given off as alarm cues, predator cues, or food cues by organisms, alter the behavioral responses of *P. clarkii* by either increasing or decreasing movement, burrow use, and time spent in the raised posture [47]. Here, we demonstrate that crayfish exposed to newt chemical cues and TTX reduce foraging behavior and that under natural conditions, TTX may positively affect stream invertebrate community diversity by stifling invasive crayfish predation.

We assessed stream invertebrate community diversity at sites with crayfish, with newts and crayfish, and with newts only. We used these data to evaluate the general impacts that crayfish had to biodiversity in this system, but also as an initial approach to determine the ecological realism of our laboratory results, recognizing that we evaluated a limited number of sites at a relatively small scale to achieve sufficient replication and establish broad conclusions. The field data show that in the site where newts co-occur with crayfish there are greater taxa counts, greater taxonomic richness, a greater number of intolerant species, and relatively higher values of diversity and stream biological integrity compared to sites with crayfish only (see Table 1). We also found that the coexistence site had a greater number of combined sensitive taxa (Ephemeroptera, Plecoptera, and Tricoptera species; EPT taxa) compared to streams with crayfish only. Some metrics were not qualitatively different between streams, but there is still a lack of species richness, intolerant species, and EPT taxa in streams with crayfish. In addition, the CSCI score was greater and the SC-IBI 3-fold higher in the coexistence site relative to sites with crayfish only. The SC-IBI offers a comprehensive assessment of spatial and temporal trends in water quality and is useful for monitoring biological community distribution which is linked to ecosystem stability [48]. The higher SC-IBI and CSCI metrics in Trancas relative to streams with crayfish and no newts indicate that the site is more biodiverse and a presumably healthier ecosystem relative to sites with crayfish only. This is reinforced by the fact that SC-IBI was greater and CSCI scores comparable between Trancas and Arroyo. One possible explanation for the differences in biodiversity scores between the coexistence and crayfish only sites could be greater habitat quality at Trancas, but CSCI accounts for abiotic data from reference sites to compute relative scores. As such, the greater CSCI value in Trancas is not likely driven only by abiotic conditions. Similarly, Trancas had greater values for some biodiversity metrics compared to Arroyo where there are no invasive crayfish, suggesting that absence of crayfish is not necessarily associated with higher biodiversity. It would also seem reasonable to argue that newts simply select higher quality habitat, but this is unlikely given that newts have extreme site fidelity, returning to the same stretch of stream each breeding period and generally to the exact same breeding pool [49]. In addition, no manual trapping occurred in Trancas during our study period but did in all other crayfish sites. Although a wetter than expected rain-year likely facilitated coexistence [21], crayfish densities greater than 1/m^2^ were still observed at all sites. Thus, our survey data provide a reasonable starting point to begin to test more broadly our laboratory results to determine if the observed stream patterns are merely coincidental, driven by environmental conditions that were not accounted for that favor newts and greater biodiversity, or related to the presence of newts, either via TTX impacting crayfish behavior or by eliciting invertebrate antipredator behaviors.

## Conclusions

Researchers are often pressed with questions that challenge the need to protect a single species. However, our results demonstrate how the conservation of newts and protection of their habitat can mitigate the negative impacts of invasive species. Freshwater ecosystems are threatened globally by the introduction and spread of *P. clarkii* whose impacts can directly and indirectly broadly lead to reduced biodiversity and exacerbate native amphibian population declines through predation, diversion of breeding habitats, and resource utilization [50]. There are limited natural deterrents of invasive crayfish and conserving freshwater streams and inherent ecosystem functioning is of major ecological importance for biodiversity, stream health, and human health [11]. Ensuring that invasive species like *P. clarkii* do not become established in ecosystems (or if present, remain at low population numbers) and limiting the ecological impacts of invasive species not only improves native species biodiversity, but in our focal system potentially promotes biodiversity by indirectly controlling and limiting the net negative ecosystem impacts of invasive crayfish.

## Methods

### Animal collection and chemical cue preparation

Adult male crayfish were collected from the Malibu Creek Watershed (Medea and Malibu Creeks, Los Angeles County, CA, USA), sexed, and measured (postorbital margin-telson: $\overline{x}$ (s.e.m.) = 9.0 cm (± 0.5), n = 58). This watershed is devoid of *T. torosa* and there are no historical records of newts in the streams where we collected. All crayfish were housed in communal containers (24.13 cm x 51.44 cm x 17.78 cm) located in a walk-in cooler. Approximately 500 mosquito larvae (*Anopheles* spp.) were gathered from an adjacent site (Newton Creek, Los Angeles County, CA, USA). All animals were maintained at a constant local stream temperature (18^o^ C) until experiments commenced.

Tree frogs (*P. cadaverina* (n = 10), *P. regilla* (n = 6)) and California newts (*T. torosa* (n = 3)) were collected from an independent watershed in the Santa Monica Mountains (Arroyo Creek, Los Angeles County, CA, USA) and transported to the laboratory individually to prepare experimental chemical cue solutions. Solutions were prepared following methods outlined in Ota et al. (2018). Briefly, the TTX solution was prepared by serial dilution to a concentration of 3.0 × 10^− 8^ moles/L from a lyophilized TTX citrate standard (Sigma Aldrich). The newt and frog chemical cue solutions were prepared by soaking animals at a ratio of 10 L per individual in carbon filtered water for 2.5 h [30] then combining the species-specific solutions. To ensure that TTX was present in the newt effluent solution and to verify concentrations were similar to our standard solution, we collected a 10 mL aliquot of the newt solution and determined with the use of a High-Performance Liquid Chromatography (HPLC) assay the presence of TTX at a concentration of 6.2 × 10^− 8^ moles/L [51]. Due to the smaller size of tree frogs, two tree frogs were soaked at a time to maintain weight to volume ratios with newts. Carbon filtered water was used as a control and did not include a buffer.

### Experimental design and data collection

#### Movement bioassay

To determine how TTX may affect crayfish movement, we performed bioassays with a combination of a control (n = 12), the TTX solution at a concentration of 3.0 × 10^− 8^ moles/L (n = 12), and chemical cue solutions for newts and frogs (*T. torosa*, n = 6; *P. regilla*, n = 6) that consisted of experimental bins (20.32 cm x 33.02 cm, W x L) filled to a depth of ~ 6.5 cm with a solution (n = 36). The frog chemical cue solution was used to account for potential confounding elements between intraguild amphibian cues and TTX. All solutions were added to bins at 18^o^ C. To reduce antagonistic behavior generally, dividers were placed between experimental bins to limit visual cues. Each bin contained a single crayfish.

Each trial ran for 30 min and was recorded with a mounted digital camera system. Each crayfish carapace was marked with a white 2 cm dot to assist with tracking movement analyses. Via video playback, a reviewer tallied the number of instances a crayfish moved. A move was counted any time the crayfish marking was entirely displaced from its original position (i.e. a move of ~ 2 cm or greater). The reviewer was blind to the treatment group during data tallying procedures.

*Feeding bioassay*: To test if TTX would affect crayfish feeding behavior, we conducted bioassays consisting of two treatments. The experimental design of these bioassays was similar to the movement experiment, except we replaced *P. regilla* with a sympatric conspecific treefrog (*P. cadaverina*) for the frog chemical cue treatment and did not include a newt chemical cue treatment. The design consisted of 20 replicates per treatment (control: n = 20; TTX: n = 20; frog: n = 20). As a proxy for native species, we provided crayfish with mosquito larvae (n = 15), which are abundant, occupy the water column of local pools, and are generally consumed by crayfish.

At the start of each trial an adult crayfish was placed in an experimental bin. A trial ran for 60 h with data collection occurring every hour for the first 12 h, every 4 h for the following 12 h, every 6 h for the next 12 h, and once every 12 h for the final 24-hour period. This temporal sampling design was selected based on preliminary crayfish feeding patterns. At each data collection point, the number of surviving mosquito larvae in each replicate were counted. All bins were numbered to prevent bias in counts by treatment.

#### Field assessments

We evaluated stream biodiversity by collecting benthic macroinvertebrates following methods by [51] and field sampling protocols detailed in [11]. We utilized the reach-wide-benthos sampling method because streams are often lentic during non-peak periods (April – October) with typical flow rates between 5 and 7 cm/s. We collected invertebrates along a 150-m reach at seven sentinel study sites in the Santa Monica Mountains, taking a sample at 15 m intervals within each stream, and combining these 11 samples to create a single stream sample. Sample collection alternated and repeated at 25%, 50%, and 75% positions from the left bank of the stream until all 11 samples were taken. To collect a sample, a D-net was extended to the stream bed, a 0.9 m^2^ area upstream demarcated and disturbed for 30 s, after which the net was removed from the water with an upstream motion. Because this method is designed for wadable streams that typically have low gradients, no specific habitat type is targeted. Newts only coexist with crayfish at one of our crayfish stream sites (Trancas) and the other four crayfish sites have no historical record of newt presence prior to crayfish introductions (Lindero Upper, Lindero Lower, Medea, MLV). At Trancas, coexistence occurs, our collection site is focused on stream habitat where newts annually breed, typically returning to the same 400 m stretch of stream each year. Post-collection, a sample was sorted, and invertebrates were enumerated and identified, typically to species level (genus when not feasible) with chironomids identified to subfamily level following statewide protocols [52].

### Statistical analyses

We analyzed the bioassay data sets independently using generalized linear mixed-effects models with Poisson distributions using the *nlme* package (v. 3.1–145, [41]) in *R* [53]. To analyze the movement behavior, we treated the number of moves as the response variable and coded the predictor for treatment (TTX, newt, frog, or control) as a fixed factor. To account for repeated measures in the feeding experiment, potential inter-individual variation, and overdispersion, we fit the models with a random effect for individual. We chose this modeling approach to account for overdispersion instead of quasi models, although we also evaluated the results of other modeling approaches that account for overdispersion, using log-normal Poisson (*glmer*), negative binomial (*glmer.nb*), and overdispersion Poisson distribution models (*glmmTMB*, family = genpois(link = log) (see Supporting Materials). Contrasts between treatments and the control were performed by recoding the reference group in our primary Poisson model in *nlme*.

To evaluate macroinvertebrate community patterns in crayfish invaded streams where newts are present or absent, we used *R* and the package *CSCI* (v. 1.2.3; [54]) to derive estimates of biotic integrity and diversity using standardized metrics for relative comparisons between streams (California Stream Condition Index (CSCI), Southern California Index of Biological Integrity (SC-IBI), S, H). The software requires habitat data for each site, including latitude and longitude, elevation, elevational range, the area of the catchment, annual average air temperatures, total precipitation and summer average precipitation, bulk soil density, soil erodibility, and average geologic phosphorous that we processed through ArcGIS following [54]. This analysis also evaluates functional groups within samples and we compare these values between sites.

### Electronic supplementary material

Below is the link to the electronic supplementary material.

Supplementary Material 1

Supplementary Material 2

## Data Availability

The datasets supporting the conclusions of this article are available in the Dryad repository, https://datadryad.org/stash/share/w72LCbAKmQD9QfZF04oGZp9PZ8r-1EwLynjOdCvAMtg.

## List of abbreviations

TTX: tetrodotoxin
GLMM: generalized linear mixed-effects model
SC-IBI: Southern California Index of Biological Integrity
CSCI: California stream condition index
BMI: benthic macroinvertebrates
EPT: ephemeropta, plecoptera, trichopteran

## References

[1] Singh JS (2002). The biodiversity crisis: a multifaceted review. Curr Sci.

[2] Cardinale BJ, Srivastava DS, Emmett Duffy J, Wright JP, Downing AL, Sankaran M, Jouseau C (2006). Effects of biodiversity on the functioning of trophic groups and ecosystems. Nature.

[3] Duffy JE, Godwin CM, Cardinale BJ (2017). Biodiversity effects in the wild are common and as strong as key drivers of productivity. Nature.

[4] Linders TEW, Schaffner U, Eschen R, Abebe A, Choge SK, Nigatu L, Mbaabu PR, Shiferaw H, Allan E (2019). Direct and indirect effects of invasive species: Biodiversity loss is a major mechanism by which an invasive tree affects ecosystem functioning. J Ecol.

[5] Didham RK, Tylianakis JM, Hutchison MA, Ewers RM, Gemmell NJ (2005). Are invasive species the drivers of ecological change?. Trends in Ecology and Evolution.

[6] Thomas CD (2010). Climate, climate change and range boundaries. Divers Distrib John Wiley Sons Ltd.

[7] Kats LB, Ferrer RP (2003). Alien predators and amphibian declines: review of two decades of science and the transition to conservation. Divers Distrib.

[8] Kerby JL, Riley SP, Kats LB, Wilson P (2005). Barriers and flow as limiting factors in the spread of an invasive crayfish (Procambarus clarkii) in southern California streams. Biol Conserv.

[9] Mooney HA, Cleland EE (2001). The evolutionary impact of invasive species. Proc Natl Acad Sci.

[10] Rodríguez C, Bécares E, Fernández-Aláez M, Fernández-Aláez C (2005). Loss of diversity and degradation of wetlands as a result of introducing exotic crayfish. Biol Invasions.

[11] Bucciarelli GM, Suh D, Lamb AD, Roberts D, Sharpton D, Shaffer HB, Fisher RN, Kats LB (2018). Assessing effects of non-native crayfish on mosquito survival. Conserv Biol.

[12] Von Holle B (2005). Biotic resistance to invader establishment of a southern Appalachian plant community is determined by environmental conditions. J Ecol.

[13] Sax DF, Brown JH (2000). The paradox of invasion. Glob Ecol Biogeogr.

[14] Azrina MZ, Yap CK, Rahim Ismail A, Ismail A, Tan SG (2006). Anthropogenic impacts on the distribution and biodiversity of benthic macroinvertebrates and water quality of the Langat River, Peninsular Malaysia. Ecotoxicol Environ Saf.

[15] Johnson RK, Wiederholm T, Rosenberg DM. Freshwater biomonitoring using individual organisms, populations, and species assemblages of benthic macroinvertebrates. Freshwater biomonitoring and benthic macroinvertebrates Chapman and Hall New York; 1993. pp. 40–158.

[16] Deborde DDD, Hernandez MBM, Magbanua FS. Benthic Macroinvertebrate Community as an Indicator of Stream Health: the Effects of Land Use on Stream Benthic Macroinvertebrates. Science Diliman; 2016;28:5-26.

[17] Hayes TB, Falso P, Gallipeau S, Stice M (2010). The cause of global amphibian declines: a developmental endocrinologist’s perspective. J Exp Biol.

[18] Quaranta A, Bellantuono V, Cassano G, Lippe C (2009). Why amphibians are more sensitive than mammals to xenobiotics. PLoS ONE.

[19] Riley SPD, Busteed GT, Kats LB, Vandergon TL, Lee LFS, Dagit RG, Kerby JL, Fisher RN, Sauvajot RM (2005). Effects of urbanization on the distribution and abundance of amphibians and invasive species in southern California streams. Conserv Biol.

[20] Miller DAW, Brehme CS, Hines JE, Nichols JD, Fisher RN (2012). Joint estimation of habitat dynamics and species interactions: disturbance reduces co-occurrence of non-native predators with an endangered toad. J Anim Ecol.

[21] Kats LB, Bucciarelli GM, Vandergon TL, Honeycutt RL, Mattiasen E, Sanders A, Riley SP, Kerby JL, Fisher RN (2013). Effects of natural flooding and manual trapping on the facilitation of invasive crayfish-native amphibian coexistence in a semi-arid perennial stream. J Arid Environ.

[22] Gamradt SC, Kats LB, Anzalone CB (1997). Aggression by non-native crayfish deters breeding in California newts. Conserv Biol.

[23] Quan AS, Pease KM, Breinholt JW, Wayne RK. 2014. Origins of the invasive red swamp crayfish (*Procambarus clarkii*) in the Santa Monica Mountains. Aquatic Invasions Regional Euro-Asian Biological Invasions Centre (REABIC), Helsinki 9: 211–219.

[24] Rosenthal SK, Stevens SS, Lodge DM (2006). Whole-lake effects of invasive crayfish (Orconectes spp.) and the potential for restoration. Can J Fish Aquat Sci.

[25] Gamradt SC, Kats LB (1996). Effect of Introduced Crayfish and Mosquitofish on California newts. Conserv Biol.

[26] California Natural Diversity Database (CNDDB). April 2023. Special Animals List. California Department of Fish and Wildlife. Sacramento, CA.

[27] Bucciarelli GM, Kats LB (2015). Effects of newt chemical cues on the distribution and foraging behavior of stream macroinvertebrates. Hydrobiologia.

[28] Derby CD, Aggio JF (2011). The neuroecology of Chemical defenses. Integr Comp Biol.

[29] Elliott SA, Kats LB, Breeding JA (1993). The use of conspecific chemical cues for cannibal avoidance in California newts (Taricha torosa). Ethology.

[30] Zimmer RK, Schar DW, Ferrer RP, Krug PJ, Kats LB, Michel WC (2006). The scent of Danger: tetrodotoxin (TTX) as an olfactory cue of Predation Risk. Ecol Monogr.

[31] Matsumura K (1995). Tetrodotoxin as a pheromone. Nature.

[32] Noguchi Y, Suzuki T, Matsutani K, Sakakibara R, Nakahigashi R, Adachi M, Nishikawa T, Abe H (2022). An almost nontoxic tetrodotoxin analog, 5, 6, 11-trideoxytetrodotoxin, as an odorant for the grass puffer. Sci Rep.

[33] Hwang PA, Noguchi T, Hwang DF (2004). Neurotoxin tetrodotoxin as attractant for toxic snails. Fish Sci.

[34] Wilson NJ, Williams CR (2014). A critical review of freshwater crayfish as amphibian predators: capable consumers of toxic prey?. Toxicon.

[35] Horner AJ, Schmidt M, Edwards DH, Derby CD (2007). Role of the olfactory pathway in agonistic behavior of crayfish, Procambarus clarkii. Invertebr Neurosci.

[36] Delgado-Morales G, Ramon F, Hernandez-Falcon J (2004). Agonistic Behaviour in Crayfish: the importance of sensory inputs. Crustaceana.

[37] Ameyaw-Akumfi C, Hazlett B (1975). Sex recognition in the crayfish Procambarus clarkii. Science.

[38] Breithaupt T, Eger P (2002). Urine makes the difference: chemical communication in fighting crayfish made visible. J Exp Biol.

[39] Fossat P, Bacque-Cazenave J, Deurwaerdere PD, Delbecque JP, Cattaert D (2014). Anxiety-like behavior in crayfish is controlled by serotonin. Science.

[40] Ota WM, Olsen B, Bucciarelli GM, Kats LB (2018). The effect of newt toxin on an invasive snail. Hydrobiologia.

[41] Pinheiro J, Bates D, DebRoy S, Sarkar D, Team RC (2018). nlme: linear and nonlinear mixed effects models. R package version 3.1–137.

[42] Kenison EK, Weldy PY, Williams RN (2017). There must be something in the water: assessing the behavioral responses of rusty crayfish (Orconectes rusticus) to fish and amphibian predator kairomones. J Ethol.

[43] Turner AM, Bernot RJ, Boes CM (2000). Chemical cues modify species interactions: the ecological consequences of predator avoidance by freshwater snails. Oikos.

[44] Hay ME (2009). Marine Chemical Ecology: chemical signals and cues structure Marine populations, Communities, and Ecosystems. Annual Rev Mar Sci.

[45] Hay ME, Duffy JE, Fenical W (1990). Host-plant specialization decreases predation on a Marine Amphipod: an herbivore in plants Clothing. Ecology.

[46] Manefield M, Rasmussen TB, Henzter M, Andersen JB, Steinberg P, Kjelleberg S, Givskov M (2002). Halogenated furanones inhibit quorum sensing through accelerated LuxR turnover. Microbiology.

[47] Hazlett B (1999). Responses to multiple Chemical Cues by the Crayfish Orconectes virilis. Behaviour.

[48] Teodosio AS, Fábio F-L (2020). Assessment of biotic integrity in streams of biological reserve of Una, Bahia, Brazil. Intern J Zool Invest.

[49] Bucciarelli GM, Green DB, Shaffer HB, Kats LB. 2016. Individual fluctuations in toxin levels affect breeding site fidelity in a chemically defended amphibian. Proceedings of the Royal Society B 2016;283:20160468 10.1098/rspb.2016.0468 PMC4892799 27194704

[50] Ficetola GF, Siesa ME, Manenti R, Bottoni L, Bernardi FD, Padoa-Schioppa E (2011). Early assessment of the impact of alien species: differential consequences of an invasive crayfish on adult and larval amphibians. Divers Distrib.

[51] Bucciarelli GM, Li A, Kats LB, Green DB (2014). Quantifying tetrodotoxin levels in the California newt using a non-destructive sampling method. Toxicon.

[52] Ode PR, Bioassessment SOP. https://www.waterboards.ca.gov/water_issues/programs/swamp/bioassessment/docs/swamp_bioassess_sop_2007.pdf.

[53] R Core Team, Core Team R. R. (2013). R: A language and environment for statistical computing.

[54] Mazor R, Ode PR, Rehn AC, Engeln M, Boyle T, Fintel E, Verbrugge S, Yang C. 2016. SCCWRP technical report #883, SWAMP-SOP-2015-0004.

