# Supplementary material for: Native amphibian toxin reduces invasive crayfish feeding with potential benefits to stream biodiversity
Source: BMC Ecol Evol. 2023 Sep 13;23:51. doi: 10.1186/s12862-023-02162-6 (PMC10498594; doi:10.1186/s12862-023-02162-6)
Supplement: Supplementary file 2 — Supplementary Material 2 [file 12862_2023_2162_MOESM2_ESM.docx]

Supporting Material

**
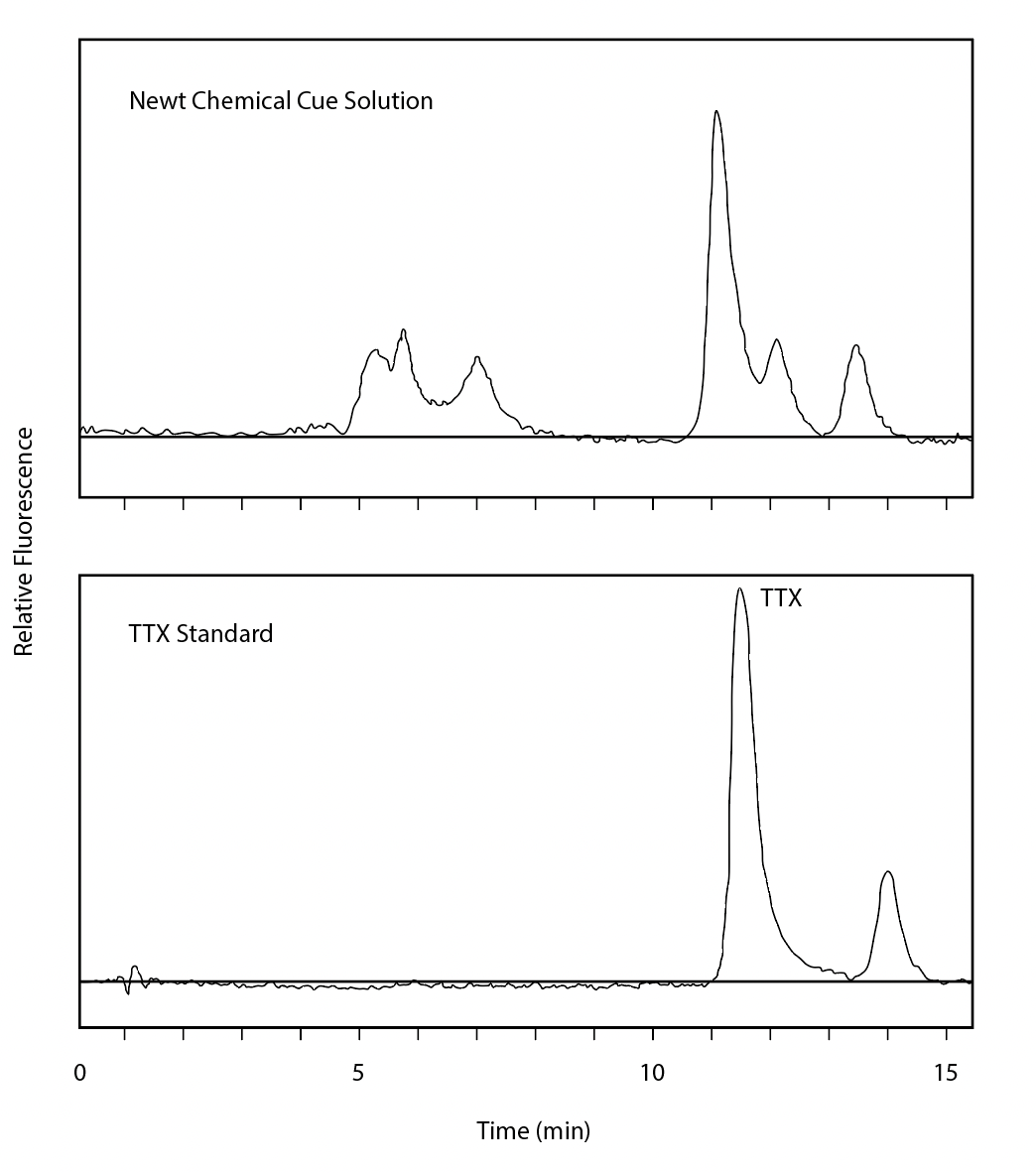
**

Supporting Figure 1: Chromatograms produced from the HPLC-FLD system show the TTX present in the newt chemical cue solution and TTX standard.

Supporting Table 1. Primary model and alternative models tested

| Survivorship Models | | β | p value |  |
| --- | --- | --- | --- | --- |
|  |  |  |  |  |
| glmer Poisson (primary) | |  |  |  |
|  | frog:control | 0.028 | 0.23 |  |
|  | newt:control | 0.119 | < 0.0001 |  |
|  |  |  |  |  |
| glmer log normal Poisson | |  |  |  |
|  | frog:control | 0.028 | 0.23 |  |
|  | newt:control | 0.119 | < 0.0001 |  |
|  |  |  |  |  |
| negative binomial | |  |  |  |
|  | frog:control | 0.028 | 0.25 |  |
|  | newt:control | 0.12 | < 0.0001 |  |
|  |  |  |  |  |
| overdispersion Poisson distribution | |  |  |  |
|  | frog:control | 0.031 | 0.223 |  |
|  | newt:control | 0.124 | < 0.0001 |  |
|  |  |  |  |  |
|  |  |  |  |  |
|  |  |  |  |  |
|  |  |  |  |  |
| Movement Models | |  |  |  |
|  |  |  |  |  |
| glmer Poisson (primary) | |  |  |  |
|  | frog:control | -0.01 | 0.95 |  |
|  | newt:control | -0.34 | < 0.01 |  |
|  | TTX:control | -0.31 | < 0.01 |  |
|  |  |  |  |  |
| glmer log normal Poisson | |  |  |  |
|  | frog:control | -0.007 | 0.95 |  |
|  | newt:control | -0.293 | < 0.01 |  |
|  | TTX:control | -0.312 | < 0.01 |  |
|  |  |  |  |  |
| negative binomial | |  |  |  |
|  | overfit (model singularity) |  |  |  |
|  |  |  |  |  |
| overdispersion Poisson distribution | |  |  |  |
|  | overfit (model singularity) |  |  |  |
